# A Quantitative Evaluation of the Influence of Chemical Variables of Biomasses of Poplar SRC Commercial Clones in Torrefaction

**DOI:** 10.3390/molecules29194542

**Published:** 2024-09-25

**Authors:** Abel Martins Rodrigues, Ana Alves, José Graça, José Rodrigues

**Affiliations:** 1 INIAV, I.P., Av. da República, Quinta do Marquês (Edifício Sede), 2780-157 Oeiras, Portugal; 2 IDMEC, Associate Laboratory LAETA, Av. Rovisco Pais 1, 1049-001 Lisboa, Portugal; 3 GeoBioTec, Largo da Torre, 2829-516 Caparica, Portugal; 4 Centro de Estudos Florestais, Laboratório Associado TERRA, Instituto Superior de Agronomia, Universidade de Lisboa, 1349-017 Lisboa, Portugal; analves@isa.ulisboa.pt (A.A.); jograca@isa.ulisboa.pt (J.G.); jocarod@isa.ulisboa.pt (J.R.)

**Keywords:** analytical pyrolysis, poplar SRC, Py-lignin, (S/G) ratio, torrefaction

## Abstract

This study aimed to evaluate the influence in torrefaction of the chemical structure of biomasses from nine poplar commercial SRC clones, evaluated through analytical pyrolysis. The chemical data were integrated into a dataset including LHV gain, representative of torrefaction aptitude and six chemical variables obtained through analytical pyrolysis, which were: (i) CH_2_Cl_2_ extractives; (ii) total extractives; (iii) Py-lignin; (iv) holocellulose; (v) (syringil/guaiacyl) ratio; and (vi) (pentosan/hexosan) ratio. Significant univariate and bivariate linear relations were obtained with LHV gain from torrefaction as dependent variable vs. Py-lignin, CH_2_Cl_2_ extractives and (cP/cH) ratio. Representative results were: (i) a negative correlation of −0.82 and −0.76 between LHV gain and the (pentosan/hexosan) ratio and Py-lignin, respectively, and (ii) a positive correlation of 0.79 between LHV gain and CH_2_Cl_2_ extractive amounts. Factorial and discriminant analysis allowed for clustering the tested clones in three groups, evidencing relevant influences of (S/G) ratio, Py-lignin, and, to a lesser extent, (cP/cH) ratio in the classification of these groups, clearly showing the impact of chemical variables of feedstock in torrefaction. The results contribute: (i) to the validation of use of the expedite analytical pyrolysis technique for classification of biomasses in accordance with their torrefaction aptitude and, thereby, (ii) to optimizing strategies in technological issues as diverse as poplar clone genetic breeding and modeling biomass torrefaction and pyrolysis.

## 1. Introduction

Under the scenario of a global increase in energy consumption of about 56% between 2010 and 2040 [1], the European Union has established as a political objective increasing the share of renewable energies to 20% of the total energy supply. Short rotation coppices (hereafter SRCs) are, in this context, a promising innovation designed for biomass production, for bioenergy that has been the subject of special attention and R&D efforts in the last 30 years. Available information on this topic includes technical field management publications [2,3], monographs on the lifecycles of SRC clusters [4], and literature on the economic feasibility of SRC cultivation from cradle to farmgate [5] as well as nutrient and biomass productivity dynamics at the plant level [6].

Biomass torrefaction is a mild pyrolysis that, by delivering a torrefied hydrophobic solid friable product with reduced (O/C) and (H/C) ratios and higher calorific power, represents a possible promising pathway of overcoming inconveniences related to low calorific power and hygroscopicity of the feedstock. Torrefaction behavior is also indicative of the aptitude of raw biomass for overall thermochemical conversion [7,8]. The chemistry behind torrefaction mainly involves the removal of oxygen and hydrogen from the biomass structure, along with an enrichment of elementary and fixed carbon [7]. In general, biomass from poplar genotypes displays average amounts of hemicelluloses, cellulose, lignin, and extractives ranging from 16% to 23%, 45% to 52%, 16% to 23%, and 0.25% to 5%, respectively [9,10]. Hemicelluloses are the main biopolymeric component to decompose and devolatilize, with xylans being prominent in this process, and their removal during torrefaction is related with the good grindability of the torrefied product [8,11]. Hexose monosaccharides are thermally more stable than pentose sugars, and the large variety of hemicellulose structures and branching allows for a large spectrum of alternative pyrolysis mechanisms. These result in similar products, although with distinct amounts [12], including furfural, ketones, and anydrosugars.

Lignin is a major amorphous polymeric component with three unit building blocks, which are p-hydroxyphenyl (H), guaiacyl (G), and syringil (S) alcohols. Due to its low % mass loss and its polymeric amorphous network structure, lignin plays a relevant role in the properties of solid torrefied products [13]. The (S/G) ratio is an index variable representative of lignin crosslinking, complementing the lignin amount, for the analysis of the dynamics of biomass biopolymers [14,15]. Although overall lower (S/G) ratios are associated with higher lignin crosslinking [9], a possibility exists that lower (S/G) ratios can be associated with thinner cell walls, making crosslinked G structures easier to degrade [15,16].

Analytical pyrolysis methodology through a micro-pyrolyzer, coupled with gas chromatography, mass spectroscopy, and flame ionization detection (Py–GC–MS/FID), allows for an accurate quantification of the composition of monomeric and polymeric units of the three major biomass components [17]. The residence time of volatiles in a micro-pyrolyzer can be as short as a few seconds, inhibiting secondary pyrolysis reactions and making the micro-pyrolyzer ideal for determining biomass composition [18]. Additionally, Py–GC/MS, by comparison of the dynamics of a micro-pyrolyzer and of a fluidized bed reactor, can contribute to a better understanding of primary and secondary pyrolysis reactional mechanisms [15]. The methodology of generalized two-dimensional perturbation correlation infrared spectroscopy (2D-PCIS), based on spectral analysis, can also be applied to the analysis of biomass pyrolysis [19,20].

Multivariate analysis techniques have been common tools for establishing correlations and validations between chemical variables of biomass obtained by instrumental pyrolytic and volumetric methodologies [21,22]. Among these techniques figure principal components or discriminant analysis [23,24]. Available information on Py–GC/MS of raw and torrefied poplar products shows that a complex mixture of compounds is formed [25], wherein compounds corresponding to phenols derived from the pyrolysis of lignin are the most abundant products. These compounds include guaiacol and vanillin. An increase in some of these phenolic compounds was indeed detected between 275 °C and 300 °C, corresponding to a concentration effect of stable structural lignin and, to a lesser extent, cellulose. This increase occurred along with a massive volatilization of biomass extractives and hemicelluloses during torrefaction [26].

Under Py–GC/FID methodology, Py-lignin is defined as the percentage ratio between the summed H, S, and G unit amounts and the sum of all pyrolysis products, including H, S, G, cP, cH, and c, wherein cP and cH refer to the pentosan and hexosan amounts, and c refers to unallocated carbohydrate compounds. The (S/G) ratio is a variable which parameterizes lignin structure [17,18]. The (cP/cH) ratio is representative of the biomass contents in cellulose and hemicellulose which are degraded under the ultrafast analytical pyrolysis with formation of 1,4-anydropentoses, e.g., arabinosans (e.g., 1,4-Anhydro-arabinofuranose) or xylosans (e.g., 1,4-Anhydro-β-D-xylofuranose), and of 1,6 anhydrohexoses, e.g., 1,6-anhydro-β-d-glucopyranose (levoglucosan-LG), the most abundant pyrolysis product from glucose, levogalactosan, or levomannosan [15]. The large variety of hemicellulose structures and branching allows for a large spectrum of alternative pyrolysis mechanisms resulting in similar products, although with distinct amounts [12], including furfural, ketones, and anydrosugars. Hexose monosaccharides are thermally more stable than pentose sugars.

The influence of chemical polymeric composition of raw biomass in torrefaction is a relevant topic, with practical implications in items such as genetic breeding and thermochemical conversion, given the main objective of biomass production attributed to poplar SRC cultivations. Few studies have focused on the impact of the polymeric structure of biomasses on their torrefaction aptitude, in comparison with those focused, for example, on the optimization of the technological process. Within the latter category, commonly assessed aspects of the torrefaction process are its mass and energy balances, its impact on the structure of biomass [27], and the comparison of types of reactors by their technologies, economic feasibility, and product quality [28].

Under the former category, three references are those of Meier et al. [23], Ru et al. [26] and Nanou et al. [29]. Ru et al. deliver a comprehensive study on the effects of torrefaction of fast-growing poplar biomass, under several operative temperatures, on biomass polymeric structure and composition, considering the main reactions occurring with chemical moieties and some physicochemical characteristics of the feedstock. These authors classify the products of Py–GC–MS analysis from this biomass in eight types of products. In particular, Type III concerns acids, especially acetic acid, a main corrosion agent derived from dissociation of O-acetyl branches from hemicelluloses; Type IV includes furfural, resulting from dehydration of xylose; and Types VII and VIII include phenols derived from lignin pyrolysis, which are the most abundant pyrolytic products. The same authors also discussed kinetic data from thermogravimetric analysis of poplar feedstock subjected to torrefaction under five temperatures between 200 °C and 330 °C, corresponding to the maximum rupture of monosaccharide rings and lignin deconstruction, with breaking of the prevailing *β*-O-4 bonds [25]. The maximum thermogravimetric weight loss rates between 250 °C and 300 °C were practically unchanged, and corresponded to a volatilization of weak moieties, leading to a concentration effect of stable lignin components in the torrefied products. On the other hand, Nanou et al. [29] discuss, for biomasses of spruce and poplar, the role of lignin components and intermediates in reactional mechanisms during torrefaction, focusing on proportions of S and G lignin units and the propensity for covalent crosslinking, along with the densification and pelletization behavior of the prevailing *β*-O-4 bonds in lignin solid torrefied product. Meier et al. [23] used analytical pyrolysis for discrimination of genetically modified poplar clones through investigation of lignin and carbohydrates and principal component analysis. These authors showed that lignin derived products were these that better discriminated the clones, and that the genetic modifications influenced the synthesis of the biopolymers in the cell walls affecting mainly the lignin moieties and carbohydrates in a lesser extent. 

Given the above context, this study aimed to evaluate the possible influence of the polymeric and extractive composition of raw woody biomasses of nine Belgian and Italian poplar SRC commercial clones, evaluated through Py–GC–MS/FID, on their torrefaction aptitude, previously reported in another study [7]. Chemical variables of raw biomasses considered for the analysis were the amounts of pentoses, hexoses, and S, G, and H lignin monomer components. The torrefaction aptitude was evaluated through methodologies of proximate, ultimate analysis, low heating value LHV, and % mass loss of the distinct raw biomasses and torrefied products [7]. These methodologies were considered proxies of the evolution of each torrefaction run as well as the chemical structure of torrefied products. The objectives of this work consisted therefore in the evaluation of whether: (i) the torrefaction performance of nine poplar SRC clones could be conveniently assessed with the chemical characterization of the biomasses by application of multivariate techniques, including discriminant analysis, relating biomass chemical and torrefaction variables; (ii) the Py–GC–MS/FID expedite methodology for biomass analysis could be a chemical analytic tool for evaluating torrefaction aptitude in accordance with objective (i).

## 2. Results and Discussion

### 2.1. Chemical Analysis of Biomass

The results of the chemical composition and of biomass torrefaction (Table 1) showed that Py-lignin varied between 22.7% (Clone Skado) and 27.1% (Clone Grimminge). The ratios (S/G) and (cP/cH) ranged between 0.78 (Clone Bakan) and 1 (Clone Hees) and 17.02% (Clone Skado) and 24.6% (Clone AF8), respectively. The cP compounds obtained from analytical pyrolysis included 1,5-anhydro-arabinofuranose and 1,5-anhydro-β-D-xylofuranose, along with levoglucosan, which was the most abundant cH product. The ratio (S/G) ranged between lower values than the referred for mature poplar species, averaging 0.9. The lower (S/G) values, typical of herbaceous biomasses, could be associated with high lignin covalent crosslinking and primary plant growth. Holocellulose amounts ranged between 68.01% (Clone AF8) and 71.38% (Clone Skado). The CH_2_Cl_2_ soluble extractive amounts ranged between 0.51% (Clone AF2) and 1.25% (Clone Skado). Water and total extractive amounts ranged between 1.3% (Clone AF2) and 2.62% (Clone Bakan) and 3.71% (Clone AF2) and 6.53% (Clone Bakan), respectively. The correlation matrix between torrefaction and chemical variables of biomass (Table 2) showed that extractives soluble in CH_2_Cl_2_ had significant correlations of −0.7, 0.79, 0.74, and 0.74 with Py-lignin, and with three torrefaction variables, which were % LHV gain, % volatile losses, and % fixed carbon gain, respectively. Furthermore, CH_2_Cl_2_ extractives presented significant correlations of 0.77 and 0.84 with water and total extractives, respectively. Indeed, this was the chemical variable with a higher number of significant correlations with torrefaction variables. Dichloromethane extracts detected were mainly composed of less polar compounds like fatty acids, alkanes, waxes, terpenes, and terpenoids. On the other hand, the (cP/cH) ratio presented significant correlations of 0.9 with Py-lignin, of −0.73 with holocellulose, and of −0.82 with LHV gain. Py-lignin showed significant correlations of −0.69 and −0.76 with holocellulose and LHV gain, respectively, besides the referred correlation with extractives in CH_2_Cl_2_ and the (cP/cH) ratio. Furthermore, Py-lignin was the variable with the most significant correlations, totaling six, with dichloromethane, water and total extractives, the cP/cH ratio, holocellulose, and LHV gain. In the same way, LHV gain was the torrefaction variable exhibiting more significant correlations with chemical variables, numbering the above-mentioned three extractives soluble in CH_2_Cl_2_, (cP/cH) ratio, and Py-lignin. Moreover, LHV gain exhibited significant correlations of 0.72 and 0.76 with % volatile losses and % fixed carbon gain, respectively, allowing it to be considered a key proxy of torrefaction efficiency. The different degrees of torrefaction and biofuel aptitude of the commercial clone biomasses, characterized essentially by higher % LHV gains, % volatile losses, oxygen, and O/C ratio % losses, were supposedly related to a higher concentration of crosslinked lignin and cellulose.

Clones Skado, Hees, and Bakan had better torrefaction aptitude, with biomasses of their torrefied products showing LHV values higher than 24 MJ·Kg^−1^ (data not shown), LHV gains higher than 30% compared with raw biomass chips, % volatile losses higher than 25%, and fixed carbon gains higher than 50% (Table 1). The good torrefaction performances of raw biomasses of clones Skado and Bakan were concomitant with higher holocellulose, lower Py-lignin amounts, and lower (S/G) and (cP/cH) ratios, tendencies reflected in Equations (1)–(4) below (Table 3). Amongst the three better torrefaction-performing clones, Hees showed the lower % mass loss and higher % volatile loss along with higher fixed carbon gain. This corresponded to higher (cP/cH) and (S/G) ratios, in comparison with biomasses of clones Skado and Bakan. The extractive amounts in dichloromethane, ethanol, and water were also relatively high in the biomasses of these three clones. Clone Wolterson followed in torrefaction aptitude, ranking well in terms of % fixed carbon gains and % volatile losses, despite a lower % LHV gain of 26.9%.

The % mass loss by devolatilization of the biomasses of the nine poplar SRC commercial clones, averaging 47%, with the release of elementary oxygen as CO_2_, formic acid, and acetic acid, corresponded mainly to the first group of pyrolysis reactions and to Type 3 compounds proposed by Ru et al. [26]. The % mass losses of biomass were compatible with solid product yields ranges of 20.3% and 29.7%, reported by Ru et al. [26], for torrefaction of fast-growing polar under operative temperatures between 250 °C and 300 °C and a residence time of 30 min. The mass losses were concomitant with the above-mentioned fixed carbon and LHV gains and high volatile losses, especially for the clones Skado, Hees, and Bakan. These losses resulted presumably from the break of ether bonds with lower activation energies and weaker thermal stability and cleaved at operative temperatures of 250 °C or higher [26], corresponding to prevailing *β*-O-4 bonds in lignin, *β*-O-1,4-glycosidic bonds in monosaccharide units of polysaccharides, or O–acetyl branches in positions C_2_ or C_3_ in hemicelluloses.

### 2.2. Exploratory Data Analysis

#### 2.2.1. Correlation and Linear Regression

**Figure 1:**
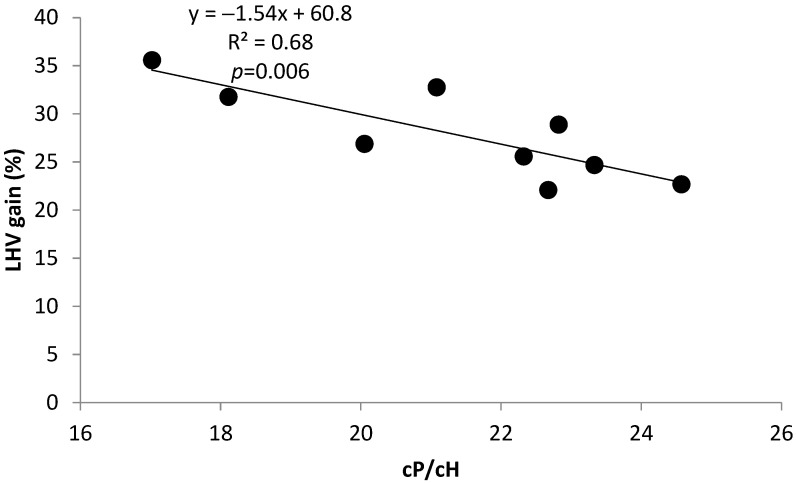
Variation of LHV gain with ratio (cP/cH).

**Figure 2:**
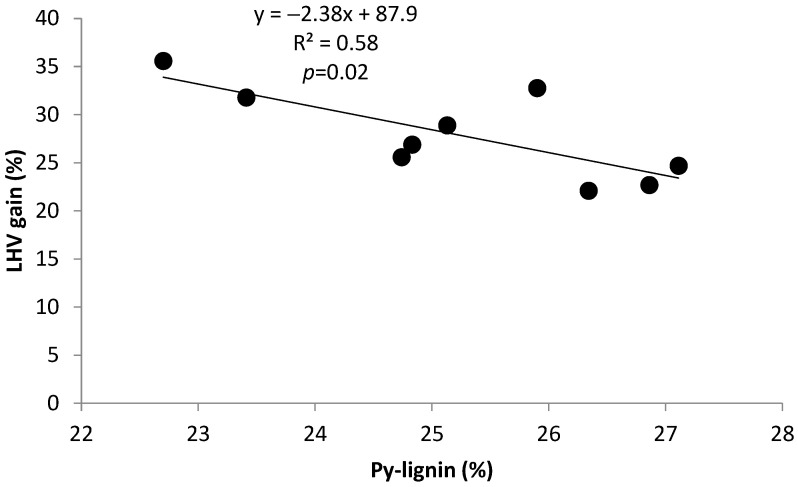
Variation of LHV gain with Py-lignin.

**Figure 3:**
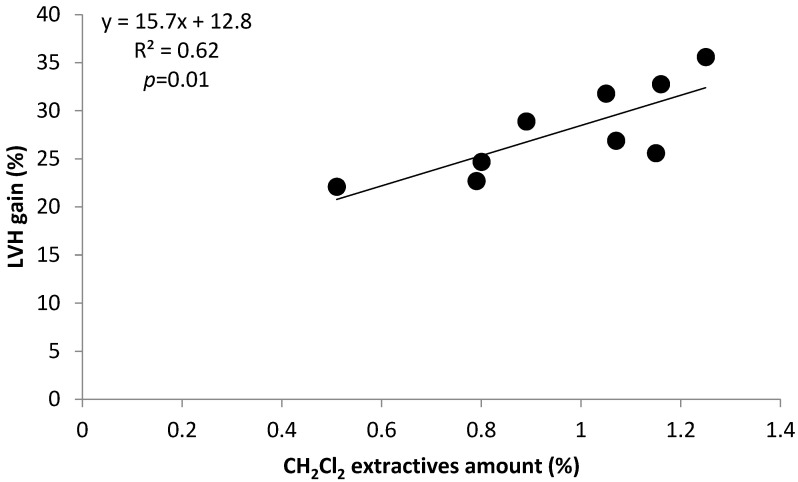
Variation of LHV gain with CH_2_Cl_2_ extractive amounts.

Significant simple linear regressions with % LHV gain, a proxy to biomass aptitude for torrefaction aptitude, as a dependent variable and (cP/cH) ratio, Py-lignin, and CH_2_Cl_2_ extractives as independent variables are displayed in the graphics in Figure 1, Figure 2 and Figure 3. The (cP/cH) ratio is representative of the monosaccharide composition of hemicelluloses and cellulose of raw biomasses. In particular, xylans are major components of SRC poplar biomass, with weight amounts as high as 22.4%. The (cP/cH) ratios of poplar clones Skado, Bakan, and Hees of 17.02, 18.11, and 21.08, respectively, were smaller than these of the other clones with lower torrefaction performance, indicating a prevalence of more thermally stable hexoses. The significant negative correlation of −0.82 between LHV gain and (cP/cH) ratio (Table 2), plotted in the linear equation in Figure 1, shows the role of thermal stable hexoses in raw biomass in the performance of torrefied products.

The negative correlation of −0.76 between Py-lignin in raw biomasses and LHV gains, (Table 2) plotted in Figure 2, presumably reflected that in the more biofuel-apt biomasses, under the seventh and eighth groups of torrefaction reactions proposed by [26], the higher predominant weaker thermally stable β-O-4 lignin bonds should devolatilize. This resulted in torrefied products with an augmented concentration of high crosslinked lignin, with higher content of C–C bonds and thus higher LHV gains.

This devolatilization of weaker lignin aryl–ether *β*-O-4 bonds enriched probably the pools of phenolic monomeric of Type 7 compounds, proposed by Ru et al. [26], like 2,6-dimethoxy-phenol or guaiacol and/or Type 8 compounds such as 2-methoxy-4-vinyl-phenol or 2,6-dimethoxy-4-(2-propenyl)-phenol. On the other hand, as mentioned, the (S/G) ratio of raw biomass lignin was relatively low, ranging between 0.78 and 1, in comparison with biomasses of other poplar species. This circumstance is associated with a higher degree of lignin covalent crosslinking and energy content of the poplar plants under a two- to three-year rotation coppice.

A contribution to biomass devolatilization during torrefaction was due to the labile extractives soluble in dichloromethane. The positive correlation of 0.79 between extractives soluble in CH_2_Cl_2_ and LHV gain (Table 2), plotted in Figure 3 and in the linear model in Table 3, reflect a contribution of these volatiles to a concentration of polymeric thermally stable polymers in torrefied products, following devolatilization. Dichloromethane extractives present in poplar species such as terpenes or phytosterols can also be recalcitrant in cell walls in wood fibers or in biomass microporosity [30,31] and resistant to thermochemical treatment up to 300 °C [26].

Bivariate significant linear regressions (1), (2), (3), and (4) in Table 3 relate LHV gain as dependent variable with three pairs of independent variables formed by CH_2_Cl_2_ extractives, Py-lignin, and (cP/cH) and (S/G) ratios, respectively. In the linear models in Table 3, the relationships between dependent and independent variables follow the tendencies expressed in the significant univariate models in Figure 1, Figure 2 and Figure 3, which show interactions between LHV gain, (cP/cH) ratio, Py-lignin, and CH_2_Cl_2_ extractives. In the linear model in Equation (3), the negative sign of the independent variable (S/G) ratio reflects the higher tendency for lignin covalent crosslinking associated with lignin with lower (S/G) ratios.

#### 2.2.2. Multivariate Data Analysis

**Figure 4:**
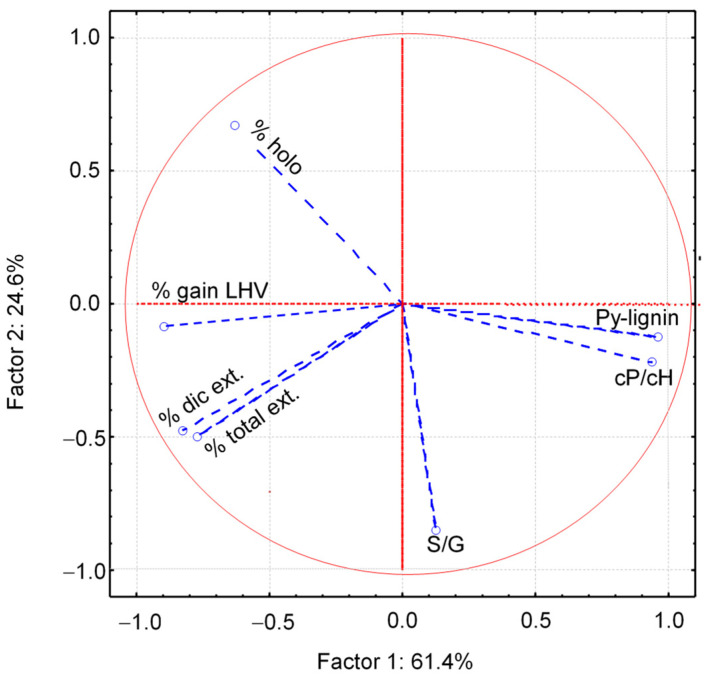
Principal component analysis: projection of chemical and LHV gain variables on the plane of Factors 1 and 2. This graph was obtained with package Statistica, 6 Statsoft.

For principal component and factorial analysis, we considered a representative matrix database of seven variables: total and CH_2_Cl_2_ extractives, LHV gain, Py-lignin, holocellulose, ratios (S/G) and (cP/cH); and nine cases: clones Skado, Bakan, Hees, Bandaris, Ellert, Grimminge, Wolterson, AF2, and AF8. The eigenvalues of the principal components of the correspondent correlation matrix are shown in Table 4, pointing to the fact that the first two principal components contributed to about 86% of the total variance. Table 5 shows the factor coordinates of variables, representing the correlations of variables with Factors 1 and 2, plotted in Figure 4. It can be seen that Py-lignin and (cP/cH) exhibit the largest positive correlations, of 0.95 and 0.93, respectively, with the first principal component, which contributes to about 61% of the total variance, while extractives in CH_2_Cl_2_ and total extractives and LHV gain correspond to the highest negative correlations with −0.83, −0.76, and −0.89, respectively.

On the other hand, the (S/G) ratio corresponds to a higher absolute negative correlation of 0.84 with the second principal component, which contributed to about 24% of the total variance. The dynamics of variable components of Factor 1, explaining about 61% of the total variance, reflect the abovementioned variability of univariate and bivariate models in Figure 1, Figure 2 and Figure 3 and Table 3.

**Figure 5:**
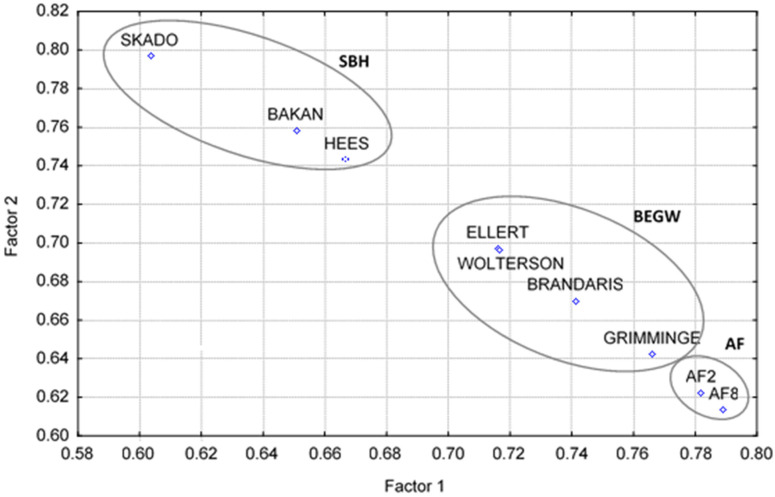
Factorial analysis: projection of factor loadings of clone as variable, with varimax rotation in the plane of Factors 1 and 2. This graph was obtained with package Statistica, 6 Statsoft. The circles with clone designations inside, resulting from the factorial analysis, are representative of the three groups of the clones, SBH, BEGW, and AF. Their location in the plane of Factor 1 and Factor 2 and clone composition reflect and cluster the distinctive torrefaction aptitudes of the nine biomasses.

The results of factorial analysis with varimax rotation applied to the matrix database are shown in Table 6 and Figure 5. Clone biomass as a whole explained about 99% of the total variability of the system. The projections of factor loadings of clone variables in the plane of the independent Factors 1 and 2 are shown in Figure 5. In this plane, clone biomasses were stratified in three groups, SBH, BEGW, and AF, corresponding to commercial clones Skado, Bakan, and Hees, with best torrefaction aptitude; Brandaris, Ellert, and Grimminge, with medium aptitude; and Wolterson and clones AF2 and AF8 with lowest torrefaction aptitude.

The projection of factor loadings in the plane of Factors 1 and 2 clearly exhibits a seriation of the clones according to their torrefaction aptitude and their chemical composition obtained by analytical pyrolysis. These factors are unobservable random variables [32] resulting from the combination of the random variables, which are LHV gain, considered a proxy for torrefaction aptitude, and the aforementioned six biomass chemical variables in the data matrix obtained from analytical pyrolysis.

Discriminant analysis for the three poplar commercial clone groups, SBH, BEGW, and AF, delivered a model with Wilk’s Lambda statistic of 0.01, indicative of a good discriminating power, with two functions (Equations (5) and (6)) with standardized coefficients, in which (S/G) ratio and Py-lignin were shown as contributive to the discrimination of the three groups:

D1 = −0.36% gain LHV − 1.99% vol loss + 1.37 (S/G) + 1.21 Py-lignin(5)

D2 = 1.33% gain LHV − 1.68% vol loss + 0.34 (S/G) + 1.63 Py-lignin(6)

**Figure 6:**
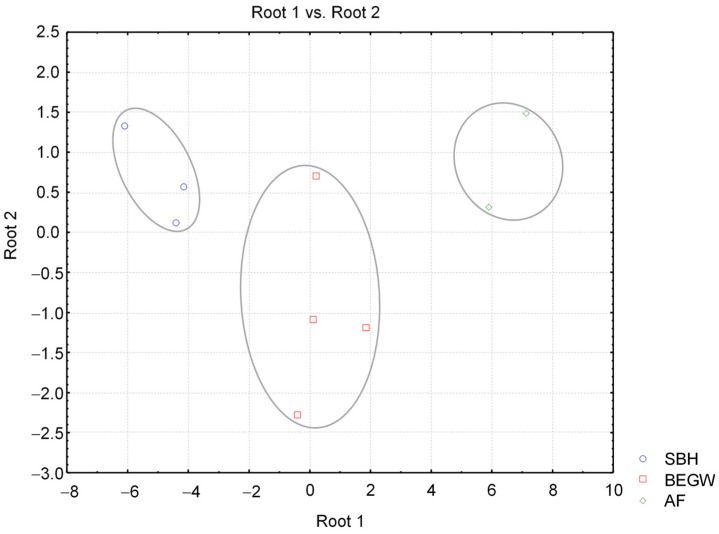
Scatter plots of canonical scores in the plane of roots 1 and 2 from discriminant analysis. This graph was obtained with package Statistica, 6 Statsoft.

The statistical analysis showed that the three clone groups were clearly separated and that % vol loss was the variable which contributed most to overall discrimination, (S/G) ratio and Py-lignin followed, and % gain LHV was the least contributing variable. Py-lignin and (S/G) ratio, pyrolytic analytical variables typical of each clone, were thereby very influential in the discrimination of the three clone groups. Scatter plots of canonical scores in the plane of roots 1 and 2 obtained by discriminant analysis are shown in Figure 6.

The three classification functions obtained from discriminant analysis and contributing to classification of biomass from an unknown commercial poplar clone in any of the three discriminated groups are given in Equations (7)–(9):

SBH = 34.63% gain LHV − 34.37% vol loss + 337.78 (S/G) + 78.07 Py-lignin − 1210.71(7)

BEGW = 32.36% gain LHV − 37.75% vol loss + 409.25 (S/G) + 81.06 Py-lignin − 1192.11(8)

AF= 32.44% gain LHV − 44.3 % vol loss + 504.37 (S/G) + 89.29 Py-lignin −1355.98(9)

Given the good discrimination power derived from a low Wilk’s Lambda statistic, it can be expected that these classification functions will be regarded as useful for applying results from analytical pyrolysis and lab torrefaction for characterizing biomass aptitude for torrefaction and thermal conversion. Overall, these functions reflect the data of higher % LHV gains, lower lignin amounts, and lower (S/G) ratios of clones from poplar clones of the three groups. All this statistical evidence clearly illustrates the usefulness of pyrolytic chemical analysis for the evaluation of the torrefaction potential of SRC woody biomasses. The variables % vol loss, (S/G) ratio, and Py-lignin were those with lower partial Wilk’s Lambda statistic values, with 0.22, 0.4, and 0.44, respectively, followed by variables % LHV gain with 0.44 and (cP/cH) (not in the model) with 0.52. These results reflected a significant influence of chemical variables of raw biomass of the poplar clones in the discrimination of biomass torrefaction aptitudes of these biomasses. From the analysis in Table 1, it can be noticed that (cP/cH) ratio distinguished commercial clones Skado, Bakan, and Hees, forming the SBH group, from the other six clones by a 1.2-fold order of magnitude, justifying therefore the mentioned relatively good influence in the overall classification.

Briefly, the matrix database obtained in this work included data from variables of torrefaction and chemical analysis by Py–GC/MS concerning raw woody biomasses and torrefied products of nine poplar SRC commercial clones. The results achieved can be useful for four main purposes, which are: (i) the validation of analytical pyrolysis as a tool for classifying poplar clones according to torrefaction aptitude; (ii) the genetic improvement of poplar genotypes through classic breeding or genetic manipulation [23,33,34,35]; (iii) the delivery of modeling insights, e.g., [25,26], about the biomass feedstock chemical structure contributing to upscaling the torrefaction to pilot and industrial scales in terms of improving the management of operative variables, such as temperature or supply storage, or of predictive modeling of mass and energy balances of the operations; and (iv) a better fundamental modeling and experimental knowledge of the composition of the mixtures of products from biomass pyrolysis, and of their potential interaction under pyrolysis operative environments [15,23,25,26].

## 3. Materials and Methods

This study consisted of the evaluation of chemical indexes in woody biomass samples by analytical pyrolysis of poplar biomass chips, with regard to variables of the composition of lignin and polysaccharides, with the objective of establishing causal and correlative relationships between biomass polymeric structure and its aptitude to torrefaction. These biomass samples were obtained from the harvesting in 2014 of seven Belgian and two Italian poplar clones cultivated as SRC in two sites, which were Lochristi in Belgium and Santarém in Portugal. The seven Belgian commercial clones and parentages [36] were: Bakan: (B: *P. trichocarpa* × *P. maximowiczii*); Brandaris: (B: *P. nigra*); Ellert: (E: *P. deltoides* × *P. nigra*); Grimmige: (G: *P. deltoides* × (*P. trichocarpa* × *P. deltoides*)); Hees: (H: *P. deltoides* × *P. nigra*); Skado: (S: *P. trichocarpa* × *P. maximowiczii*); and Wolterson: (W: *P. nigra*). The two Italian commercial clones and parentages [37] were: AF2: (*P. deltoides* × *P. nigra*) and AF8: (*P. generosa* × *P. trichocarpa*).

### 3.1. Extractives Content

The chemical procedure for biomass characterization, concerning the lignin and polysaccharide structure, involved three previous successive volumetric extractions of biomass samples for the removal and quantification of extractives. Aliquots of biomass samples (1 g) were sequentially extracted (125 mL) with dichloromethane (6 h) from Fisher Chemical (Germany), 96% ethanol (16 h) from AGA (Lisboa, Portugal), and distilled water (16 h), using a Soxhlet apparatus with 3 samples per Soxhlet. The samples were kept individually in ANKOM filter bags (ANKOM Technology, New York, NY, USA) prior to analysis. The extractives content was assessed by the weight loss after each step.

### 3.2. Analytical Pyrolysis and Torrefaction Procedures

The extracted biomass samples were thereafter subjected to analytical pyrolysis assays carried out with a CDS Pyroprobe 1000 (CDS Analytical LLC, Oxford, PA, USA) with a curl filament connected to Agilent 6890 GC (Agilent Technologies, Santa Clara, CA, USA) by a heated interface (270 °C). Samples of 74–77 μg were pyrolyzed at 600 °C for 5 s. The GC operative conditions were: injector 270 °C, detector 270 °C, pyrolytic temperature programming with a 4 min isothermal period at 45 °C followed by heating to pyrolysis temperature through a heating rate ranging between 4° and 270 °C min^−1^. Two biomass samples were previously analyzed with four replicates on different days for investigation of the precision of the Py–GC–MS/FID method, based on the pooled standard deviation of replicates. The precision was shown as high, with std of cP/cH = 0.7, H/G = 0.005, S/G = 0.03, and Py-lignin = 0.3%. Thus, the adopted procedure was of realization of one Py–GC/MS analysis by biomass sample.

The carbohydrate peaks included hexosans (cH), pentosans (cP), and unassigned carbohydrate (c). The Py-lignin is defined as (H + G+S)/(H + G+S + c+cP + cH), where the numerator is the sum of peak areas of the three lignin phenylpropane units (H, G, and S) obtained by integration with the software Chemstation (Agilent Technologies, Palo Alto, Santa Clara, CA, USA), normalized to percentages. Holocellulose amounts, including cellulose and hemicellulose, were obtained through the difference between the total biomass and the amounts of lignin and extractives. A detailed description of the whole analytical pyrolysis procedure is presented in Rodrigues et al. [17] and Alves et al. [18,24,38].

For obtaining torrefied products, biomass samples of about 1 kg were subjected to a lab torrefaction assay at a nitrogen atmosphere in a laboratorial Nabertherm rotary reactor, under an operative temperature of 265 °C, residence time of 15 mins, and a total 1 h 45 min heating period, as described in Rodrigues et al. [9]. LHV of as-received biomass and torrefied products was determined in the laboratory with a Parr 6400 calorimeter (Parr Instrument Company, Moline, IL, USA), based on standard EN 14918 [39] and on the equipment’s operating instructions. Proximate of biomass and torrefied products was determined in laboratory with a Thermogravimetric Analyzer ELTRA thermostep (ELTRA GmbH, Haan, Germany), following the standard ASTM D7582-12 [40]. Ultimate analysis of feedstocks was carried out with a LECO CHN628 (Leco Corporation, Michigan, MI, USA) elemental analyzer following standard operating instructions.

The variables considered in both feedstocks were fixed carbon, volatiles, ash amount, elementary carbon (C), elementary oxygen (O), elementary hydrogen (H), and (O/C) and (H/C) ratios. The impact of torrefaction on the structure of biomass was additionally assessed through carbon gain, % mass loss, % oxygen loss, and % volatile loss [9].

The torrefaction and analytical pyrolysis data were grouped in a matrix database, including variables: (i) extractables in dichloromethane, (ii) extractables in ethanol, (iii) extractables in water, (iv) total extractables, (v) (pentose/hexose) ratio (cP/cH), (vi) (syringyl/guaiacyl) (S/G) ratio, (vii) Py-lignin, (viii) (H/G) ratio, (ix) holocellulose (holoPy), (x) LHV, (xi) LHV gain, (xii) volatile % loss, (xiii) fixed carbon % gain, (xiv) % (O/C) loss, (xv) % carbon gain, (xvi) % mass loss, and (xvii) % oxygen loss.

### 3.3. Statistical Analysis

Analytical exploratory statistical calculations were carried out with the package Statistica, version 6 Statsoft, for relating these variables with an assessment of latent tendencies among them, and seriation and classification of poplar clones according to their torrefaction aptitude. The statistical analysis involved correlation analysis for searching of significant univariate and multivariate regressions, through a general linear model, principal components, and matrix factorial analysis without and with varimax rotations and discriminant analysis, delivering discriminant and classification functions. Discriminant analysis was performed over three groups of case variables, SBH, BEGW, and AF, corresponding to categorization of the clones as best, medium, and least apt for torrefaction conversion. The significance of the discriminating power between these groups, and the influence of individual variables on the classification power of clones in the same groups, were respectively evaluated through computation of Wilk’s Lambda and partial Wilk’s statistics.

## 4. Conclusions

The results of this study contribute to validating the use of expedite analytical pyrolysis as a tool for evaluating the torrefaction aptitude of biomasses from commercial SRC poplar clones. A matrix dataset was established including LHV gain, representative of torrefaction aptitude, and six chemical variables obtained through analytical pyrolysis, which were: (i) CH_2_Cl_2_ extractives; (ii) total extractives; (iii) Py-lignin; (iv) holocellulose; (v) (syringil/guaiacyl) ratio; and (vi) (pentosan/hexosan) ratio. From this matrix, three significant univariate and four bivariate linear significant models were extracted, with LHV gain as dependent variable and Py-lignin, CH_2_Cl_2_ extractives, and (pentosan/hexosan) ratio as independent variables. These models reflected the (i) negative correlations between LHV gain and the (pentosan/hexosan) ratio and Py-lignin, and (ii) a positive correlation between LHV gain and CH_2_Cl_2_ extractive amounts. Factorial and discriminant analysis allowed for clustering the tested clones in three groups, evidencing relevant influences of (S/G) ratio, Py-lignin, and, to a lesser extent, (pentosan/hexosan) ratio in the classification of these groups, clearly showing the impact of chemical variables of feedstock in torrefaction. The results obtained can be useful for the genetic improvement of poplar genotypes, for the knowledge of the impact of biomass feedstock chemical structure on the modeling of upscaling of torrefaction operations, and for the fundamentals of the potential interactions of products from biomass pyrolysis under pyrolytic dynamics.

## Figures and Tables

**Table 1:** Chemical analysis of woody biomasses from nine poplar SRC commercial clones.

Clone	% CH_2_Cl_2_ Extractives	% ethanol Extractives	% H_2_O Extractives	% Total Extractives		(cP/cH) Ratio	(S/G) Ratio	Py-Lignin (%)	(H/G) Ratio
Skado	1.25	2.41	2.26	5.92		17.02	0.85	22.70	0.13
Bakan	1.05	2.86	2.62	6.53		18.11	0.78	23.41	0.20
Hees	1.16	2.62	2.04	5.83		21.08	1.00	25.90	0.12
Brandaris	1.15	2.71	2.47	6.33		22.32	0.94	24.74	0.15
Ellert	0.89	2.23	1.89	5.01		22.82	0.94	25.13	0.10
Grimminge	0.80	1.53	1.44	3.77		23.33	0.79	27.11	0.09
Wolterson	1.07	2.51	1.67	5.26		20.05	0.99	24.83	0.11
AF8	0.79	2.57	1.76	5.13		24.57	0.96	26.86	0.14
AF2	0.51	1.90	1.30	3.71		22.67	0.85	26.34	0.16
Clone	% HoloPy	LHV (MJkg^−1^) (*)	% LHVGain (#)	% Volatiles Loss (#)	% Fixed Carbon Gain (#)	% Ratio (O/C) Loss (#)	% Carbon gain (#)	% Mass Loss Torrefaction (#)	% Oxygen Loss (#)
Skado	71.38	18.48 ± 0.4	35.60	25.37	51.05	51.53	30.62	46.83	36.68
Bakan	70.06	18.53 ± 0.6	31.79	23.90	50.65	47.63	27.96	49.58	32.98
Hees	68.27	18.80 ± 0.9	32.78	29.90	53.16	50.80	29.92	45.14	36.03
Brandaris	68.93	18.70 ± 0.6	25.60	22.30	45.08	49.60	28.65	43.21	35.19
Ellert	69.86	18.70 ± 0.7	28.90	23.90	48.30	52.42	30.89	46.60	37.73
Grimminge	69.12	18.60 ± 1.1	24.70	24.70	48.48	52.80	30.84	49.77	38.30
Wolterson	69.92	18.70 ± 0.4	26.90	25.60	53.02	40.06	30.62	47.81	26.67
AF8	68.01	18.50 ± 0.6	22.70	21.04	44.40	48.67	28.74	45.80	33.92
AF2	69.96	18.50 ± 0.7	22.11	17.30	41.30	43.45	24.52	45.80	29.58

(*) LHV refers to raw biomass; (#) percent variables calculated from average values of the concerned variables.

**Table 2:** Correlations between chemical, proximate and ultimate analysis, and LHV of nine poplar SRC commercial clones.

	CH_2_Cl_2_ Extr.	Ethanol Extr.	H_2_O Extr.	Total Extr.	cP/cH	S/G	Py-Lignin	H/G	HoloPy	LHV	Gain LHV
CH_2_Cl_2_ extr.	1.00	0.65	0.77	0.84	−0.67	0.24	−0.70	0.01	0.29	0.38	0.79
Ethanol extr.	0.65	1.00	0.79	0.92	−0.42	0.40	−0.55	0.56	−0.08	0.19	0.43
H_2_O extr.	0.77	0.79	1.00	0.95	−0.58	−0.05	−0.75	0.53	0.39	0.09	0.64
Total extr.	0.84	0.92	0.95	1.00	−0.58	0.20	−0.73	0.47	0.21	0.21	0.64
cP/cH	−0.67	−0.42	−0.58	−0.58	1.00	0.28	0.90	−0.34	−0.73	0.15	−0.82
S/G	0.24	0.40	−0.05	0.20	0.28	1.00	0.19	−0.32	−0.62	0.64	−0.05
Py-lignin	−0.70	−0.55	−0.75	−0.73	0.90	0.19	1.00	−0.40	−0.69	0.14	−0.76
H/G	0.01	0.56	0.53	0.47	−0.34	−0.32	−0.40	1.00	0.20	−0.47	0.08
HoloPy	0.29	−0.08	0.39	0.21	−0.73	−0.62	−0.69	0.20	1.00	−0.16	0.41
LHV	0.38	0.19	0.09	0.21	0.15	0.64	0.14	−0.47	−0.16	1.00	0.15
Gain LHV	0.79	0.43	0.64	0.64	−0.82	−0.05	−0.76	0.08	0.41	0.15	1.00
Volatiles loss	0.74	0.27	0.33	0.43	−0.41	0.30	−0.24	−0.40	−0.02	0.62	0.72
Fixed carbon gain	0.74	0.32	0.35	0.46	−0.63	0.17	−0.45	−0.27	0.15	0.45	0.76
Carbon gain	0.59	0.02	0.16	0.22	−0.16	0.24	−0.17	−0.69	0.00	0.43	0.46
			Volatiles Loss			Fixed Carbon Gain			Carbon Gain		
CH_2_Cl_2_ extr.			0.74			0.74			0.59		
Ethanol extr.			0.27			0.32			0.02		
H_2_O extr.			0.33			0.35			0.16		
Total extr.			0.43			0.46			0.22		
cP/cH			−0.41			−0.63			−0.16		
S/G			0.30			0.17			0.24		
Py-lignin			−0.24			−0.45			−0.17		
H/G			−0.40			−0.27			−0.69		
HoloPy			−0.02			0.15			0.00		
LHV			0.62			0.45			0.43		
Gain LHV			0.72			0.76			0.46		
Volatiles loss			1.00			0.92			0.76		
Fixed carbon gain			0.92			1.00			0.71		
Carbon gain			0.76			0.71			1.00		

**Table 3:** Significant linear relations between LHV gain, CH_2_Cl_2_ extractive amounts, Py-lignin, (cP/cH) and (S/G) ratios, and holocellulose.

Y = 50.2 + 10.1 x_1_ − 1.3 x_2_; R^2^ = 0.70	*p* = 0.02	Y: LHVgain; x_1_: ext. CH_2_Cl_2_; x_2_: Py-lignin	(1)
Y = 41.0 + 8.6 x_1_ − 1.0 x_2_; R^2^ = 0.78	*p* = 0.01	Y: LHVgain; x_1_: ext. CH_2_Cl_2_; x_2_: (cP/cH) ratio	(2)
Y = 23.9 + 16.9 x_1_ − 13.8 x_2_; R^2^ = 0.68	*p* = 0.03	Y: LHVgain; x_1_: ext. CH_2_Cl_2_; x_2_: (S/G) ratio	(3)
Y = 65.0 − 0.3 x_1_ − 1.4 x_2_; R^2^ = 0.68	*p* = 0.03	Y: LHVgain; x_1_: Py-lignin; x_2_: (cP/cH) ratio	(4)

**Table 4:** Principal component analysis: eigenvalues of correlation matrix.

	Eigenvalue	% Total Variance	Cumulative Eigenvalue	Cumulative % Variance
1	4.29	61.39	4.29	61.39
2	1.71	24.48	6.01	85.88
3	0.47	6.82	6.48	92.71
4	0.31	4.46	6.80	97.17
5	0.11	1.59	6.91	98.77
6	0.08	1.22	7.00	100.0

**Table 5:** Principal component analysis: factor coordinates of variables, considering the first two principal components.

	Factor 1	Factor 2
%CH_2_Cl_2_	−0.83	0.47
%Total	−0.76	0.50
LHV gain	−0.89	0.09
Py-lignin (%)	0.95	0.12
HoloPy	−0.63	0.67
S/G	0.12	−0.84
cP/cH	0.93	−0.21

**Table 6:** Factorial analysis of clone as variables: factor loadings of variables, with varimax rotation.

	Factor 1	Factor 2
Skado	0.60	0.79
Bakan	0.65	0.75
Hees	0.66	0.74
Brandaris	0.74	0.67
Ellert	0.71	0.69
Grimminge	0.76	0.64
Wolterson	0.71	0.69
AF8	0.78	0.61
AF2	0.78	0.62
Expl.Var	4.63	4.36
Prop.Total	0.51	0.48

## Data Availability

The data presented in this study are included in the article.
